# *Enterobacteriaceae* and *Bacteroidaceae* provide resistance to travel-associated intestinal colonization by multi-drug resistant *Escherichia coli*

**DOI:** 10.1080/19490976.2022.2060676

**Published:** 2022-04-07

**Authors:** Matthew Davies, Gianluca Galazzo, Jarne M. van Hattem, Maris S. Arcilla, Damian C. Melles, Menno D. de Jong, Constance Schultsz, Petra Wolffs, Alan McNally, Willem van Schaik, John Penders

**Affiliations:** a Institute of Microbiology and Infection, University of Birmingham, Birmingham, UK; b Department of Medical Microbiology, School of Public Health and Primary Care (CAPHRI), Maastricht University Medical Centre (MUMC+), Maastricht, The Netherlands; c Department of Medical Microbiology, School of Nutrition and Translational Research in Metabolism (NUTRIM), Maastricht University Medical Centre (MUMC+), Maastricht, The Netherlands; d Department of Medical Microbiology, Amsterdam University Medical Center, AMC, Amsterdam, The Netherlands; e Department of Medical Microbiology and Infectious Diseases, Erasmus University Medical Centre, Rotterdam, The Netherlands; f Department of Global Health, Amsterdam Institute for Global Health and Development, AMC, Amsterdam, The Netherlands

**Keywords:** Extended-spectrum beta-lactamase, travel, microbiome, antimicrobial resistance, metagenome

## Abstract

Previous studies have shown high acquisition risks of extended-spectrum beta-lactamase-producing *Enterobacteriaceae* (ESBL-E) among international travelers visiting antimicrobial resistance (AMR) hotspots. Although antibiotic use and travelers’ diarrhea have shown to influence the ESBL-E acquisition risk, it remains largely unknown whether successful colonization of ESBL-E during travel is associated with the composition, functional capacity and resilience of the traveler’s microbiome. The microbiome of pre- and post-travel fecal samples from 190 international travelers visiting Africa or Asia was profiled using whole metagenome shotgun sequencing. A metagenomics species concept approach was used to determine the microbial composition, population diversity and functional capacity before travel and how it is altered longitudinally. Eleven travelers were positive for ESBL-E before travel and removed from the analysis. Neither the microbial richness (Chao1), diversity (effective Shannon) and community structure (Bray–Curtis dissimilarity) in pretravel samples nor the longitudinal change of these metrics during travel were predictive for ESBL-E acquisition. A zero-inflated two-step beta-regression model was used to determine how the longitudinal change in both prevalence and abundance of each taxon was related to ESBL acquisition. There were detected increases in both the prevalence and abundance of *Citrobacter freundii* and two members of the genus *Bacteroides*, in association with remaining uncolonized by ESBL-E. These results highlight the potential of these individual microbes as a microbial consortium to prevent the acquisition of ESBL-E. The ability to alter a person’s colonization resistance to a bacterium could be key to intervention strategies that aim to minimize the spread of MDR bacteria.

## Introduction

International travel has increased in parallel with globalization, with pre-SARS-CoV-2 pandemic statistics of over 1 billion tourist arrivals occurring each year.^1^ Trafficking between countries with varying degrees of AMR has resulted in the substantial spread of multidrug-resistant (MDR) bacteria across the globe.^2–6^ In particular, travel to destinations in South(eastern) Asia and Northern Africa has been linked to a high risk of acquisition of MDR bacteria. For example, up to 84% of travelers to India and 42% of travelers to Morocco have been shown to have acquired MDR *Enterobacteriaceae* (MDR-E) in their intestines upon travel return.^7^ Considering that the intestinal tract is an open system, which is confronted with a myriad of bacteria from the environment (e.g. food, water, soil, other humans or animals), the human intestinal microbiota is considered the most important reservoir through which travelers acquire and import AMR.^8^

Although most travelers will lose the acquired MDR strains within months after their return from travel, the acquisition of intestinal MDR bacteria can pose a direct health threat to the traveler. Specifically, when hospitalized, most significantly within intensive care units that frequently use large amounts of antibiotics,^9^ or when undergoing specific medical procedures,^10^ intestinal colonization by MDR bacteria substantially increases the risk of MDR infections and consequently mortality. *Enterobacteriaceae* are the cause of some of the most common nosocomial MDR infections,^11^ and with the high rates of acquisition of Extended Spectrum Beta Lactamase-producing *Enterobacteriaceae* (ESBL-E) during international travel, the admission of travelers to hospitals is posing a serious health risk. Moreover, the introduction of MDR bacteria by international travelers also poses a potential risk for public health, as this permits the transfer of MDR bacteria to household members.^12^ Identifying successful intervention strategies to prevent MDR-E acquisition by travelers would therefore not only directly protect vulnerable groups of travelers that have an increased risk of infections by (translocation of) intestinal (MDR) bacteria (e.g., immunocompromised individuals or patients undergoing surgery) but also reduce the spread of AMR across the globe.

Besides travel destination, the use of antibiotics during travel^2,7,13^ and travelers’ diarrhea^2,7,13–15^ have been repeatedly shown to increase the risk of MDR-E among travelers. In addition, travelers with pre-existing chronic bowel disease have a higher risk of acquiring MDR-E.^7^ Each of these factors are linked to major perturbations to the gut microbiome, suggesting that the microbiome might play a pivotal role in the susceptibility to MDR-E colonization upon travel. Indeed, the role of the intestinal microbiome in providing colonization resistance against incoming opportunistic pathogens has long been established.^16^ Endogenous microbes can directly or indirectly interfere with pathogen colonization through a variety of mechanisms, including the competition for nutrients and the production of small antimicrobial peptides (bacteriocins) .^17–19^ Moreover, the stimulation of the host immune system by the commensal microbiome has been shown to interfere with pathogen colonization.^20^

To uncover the effect of travel on the gut microbiome, a previous study employed metatranscriptomics on fecal samples from 43 travelers visiting tropical regions.^21^ No significant associations between the intestinal microbiota composition before or after travel and the risk of MDR-E acquisition was found. However, the microbiota profiles did significantly differ between individuals who cleared MDR-E within one month and those who were persistently colonized. In another study among 10 international travelers, profiling of longitudinally collected fecal samples by metagenomic next-generation sequencing did not also reveal alterations in microbiome diversity and composition in those acquiring MDR-E.^22^

These inconclusive results highlight the need for a larger-scale longitudinal study using whole metagenome sequencing (WMGS) to examine if specific microbial taxa and their functional capacities may protect against MDR-E acquisition during travel.

Here, we performed WMGS on fecal swabs of 190 travelers before travel and immediately upon return from South Asia, Southeast Asia, North Africa and East Africa. Metagenome sequences were processed via coabundance gene clustering to create taxonomic and metabolic profiles. In order to identify taxa and traits associated with a reduced rate of MDR-E acquisition, the (dynamics in) microbiome community structure, composition and functional profile were compared between travelers who acquired MDR-E during travel and those that did not.

## Results

Fecal culturing revealed that 103 (57.5%) out of the 179 travelers who were negative for ESBL-E prior to travel acquired ESBL-E during their trip. From the post-travel fecal samples (T1) of these 103 travelers, a total of 148 morphologically distinct ESBL-E strains were isolated. The vast majority of strains were identified as *E. coli* (136/148, 91.9%), 9 strains (6.1%) were characterized as *Klebsiella pneumoniae* and the remaining 3 strains were characterized as *Proteus mirabilis, Citrobacter freundii* and *Klebsiella ornithinolytica*.

Fecal swabs were whole metagenome sequenced and processed into coabundance genomes (CAGs; see methods). Upon quality filtering and preprocessing of WMGS data of the pre- and post-travel samples, 37,381 CAGs were generated of which 144 CAGs contained more than 700 genes and were classified as MGS.

### Baseline microbiome is not significantly associated with risk of acquiring ESBL-E

**Figure 1.**
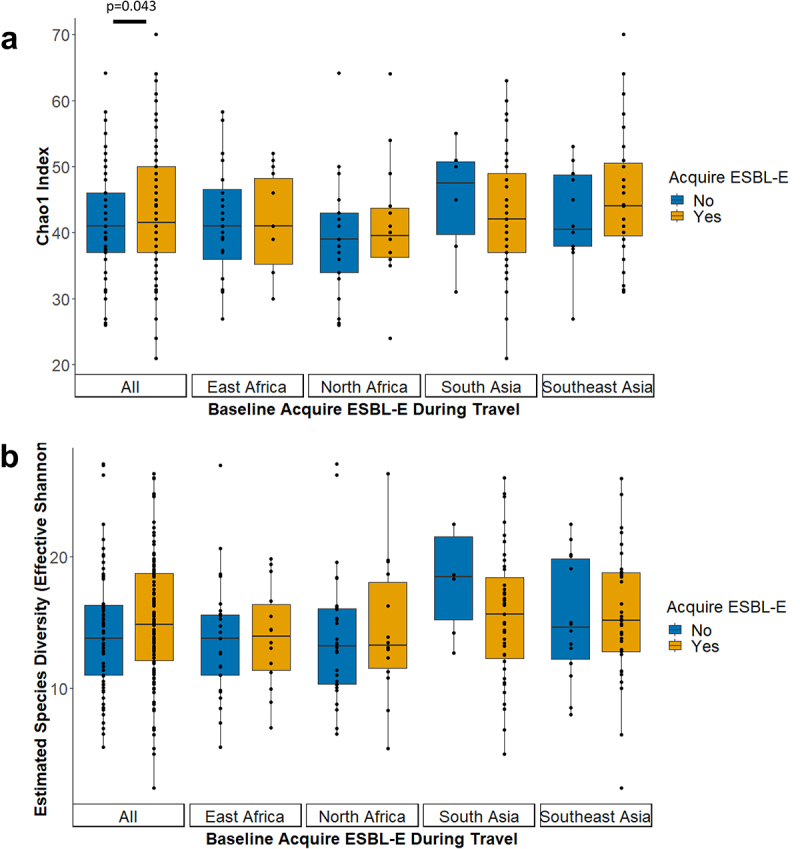
Baseline microbial species richness and diversity in association with ESBL-E acquisition. (A) Estimated (Chao1) species richness and (B) microbial diversity (effective Shannon) in fecal samples collected at baseline. Only metagenomic species (coabundance gene groups with >700 genes) were included in the diversity calculations. Differences in baseline alpha diversity metrics between individuals that did or did not acquire ESBL-E were tested using linear regression analyses, and significant differences are visualized with their p-values. To adjust for potential confounding factors, alpha diversity metrics were treated as dependent variables and ESBL-E acquisition, sex, age, BMI and travelers’ diarrhea as independent variables, to produce coefficient estimates, standard errors and p-values (Supplementary Table 1).

The ability of the baseline microbiome to predict a traveler’s risk of acquiring ESBL-E was determined by the richness, diversity, community structure and taxonomic composition of the MGS observed in pretravel samples of travelers who did or did not subsequently acquire ESBL-E during travel. The observed number of metagenomic species (Species Richness; Supplementary Table S1) and estimated number of species (Chao1; Figure 1A) were significantly higher (Chao1 coefficient estimate: 2.82, 95% CI [0.09, 5.56]) in baseline fecal samples of travelers who acquired ESBL-E than those who did not acquire ESBL-E, as determined using linear regression analysis. When stratifying the analyses according to travel destination, these differences were, however, no longer observed. In contrast, the effective number of species as calculated from Shannon diversity (Figure 1B) was neither statistically significantly associated with ESBL-E acquisition in the overall study population (*P* = .235) nor when stratified to travel destination (Effective Shannon, Supplementary Table S1).

**Figure 2.**
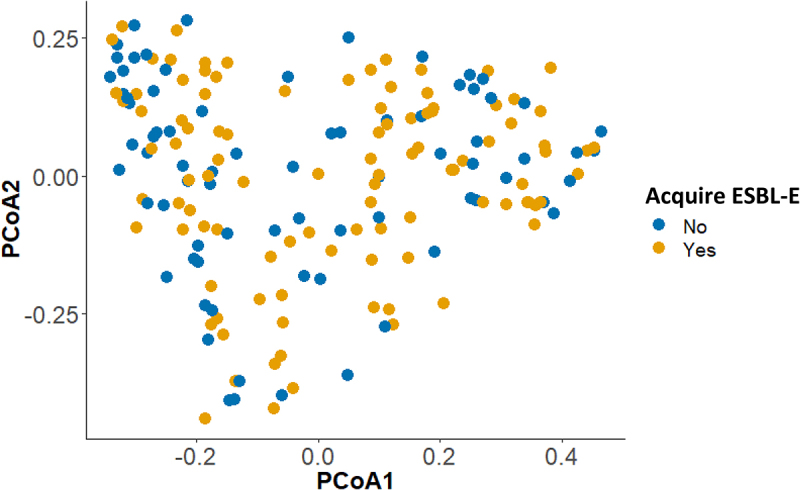
Baseline microbial community structure (Bray-Curtis) in association with subsequent ESBL-E acquisition during travel. Ordination was performed using PCoA based on Bray–Curtis dissimilarity. Only metagenomic species (coabundance gene groups with >700 genes) were included in the diversity calculations. Samples are colored according to subsequent ESBL-E acquisition during travel.

We subsequently examined whether the overall microbial community structure prior to travel, as examined by means of the Bray–Curtis dissimilarity, was associated with ESBL-E acquisition risk. Ordination of pretravel samples in Principal Coordinates Analyses (PCoA) did, however, not show any clustering according to ESBL-E acquisition. Fitting the ESBL-E acquisition status to the ordination, using envfit, confirmed the lack of association between the baseline microbial community structure and the risk of subsequently acquiring ESBL-E during travel (figure 2; P = 3).

The significance of age, BMI, sex and ESBL-E acquisition in the microbial community structure was determined using the envfit function in vegan. The significance value was determined based on 999 permutations. All P values derived from envfit were adjusted for multiple comparisons using FDR adjustment (Benjamini–Hochberg procedure) (Supplementary Table 2).

***Microbiome structure and diversity are altered during travel, but mainly associated with the development of travelers’ diarrhea and not the acquisition of ESBL-E***

**Figure 3.**
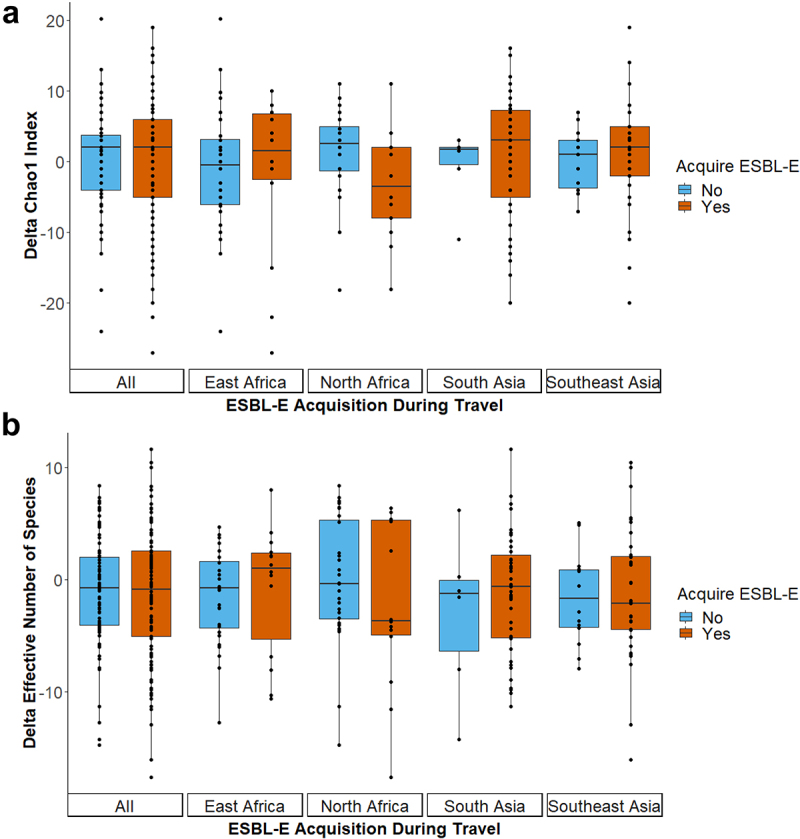
Stability in microbial species richness and diversity in association with ESBL-E acquisition. The delta between the (A) estimated species richness (Chao1) and (B) microbial diversity (effective Shannon) between pre- and post-travel fecal samples was calculated for each individual. Only metagenomic species (coabundance gene groups with >700 genes) were included in the diversity calculations. Differences in stability of species richness and diversity between individuals who did or did not acquire ESBLs were tested using linear regression analysis. To adjust for potential confounding factors, analyses were performed with delta in alpha diversity metrics as dependent variables and ESBL-E acquisition, sex, age, BMI, travelers’ diarrhea and consumption of food from street food stalls as independent variables (Supplementary Table 1).

To examine the association between microbiome stability and the susceptibility to ESBL-E acquisition, longitudinal analyses were performed. When examining the shifts in alpha diversity metrics within travelers, the estimated microbial richness (Figure 3A) and microbial diversity (Figure 3B) were not associated with ESBL-E acquisition (*P* = .95 and *P* = .85, respectively). Also when stratifying the analyses for travel destination, no associations between these measurements and ESBL-E acquisition were observed during travel (Supplementary Table 1).

However, a statistically significant decrease in the microbial richness (coefficient estimate: −4.47, 95% CI [−6.82, −2.14]) and diversity (coefficient estimate: −3.47, 95% CI [−5.05, −1.91]) was associated with travelers’ diarrhea (both *P* < .001). When stratified for travel destination, the negative association with diarrhea was still observed for travelers visiting East Africa and South Asia (Supplementary Table 1).

**Figure 4.**
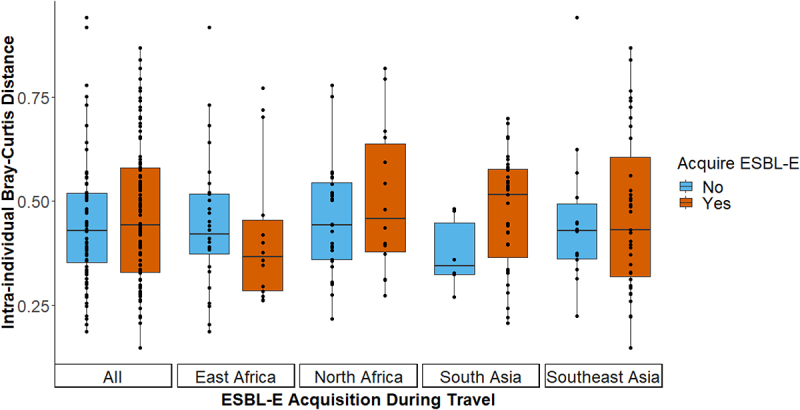
Stability in the microbial community structure in association with ESBL-E acquisition. Intraindividual Bray–Curtis dissimilarity between pre- and post-travel samples. Differences in stability in the microbial community structure between individuals who did or did not acquire ESBLs were tested using linear regression analyses. Analyses were performed with intraindividual Bray–Curtis dissimilarity as the dependent variable and ESBL-E acquisition, sex, age, BMI, travelers’ diarrhea and consumption of food from street food stalls as independent variables (Supplementary Table 2).

As for the stability in microbial richness and diversity, the stability in the microbial community structure was also examined among travelers. The within-subject Bray–Curtis distance between pre- and post-travel samples was not significantly different between travelers who did or did not acquire ESBL-E (figure 4; *P* = .504). Upon stratification according to travel destination, this lack of significant differences in the stability of the microbial community structure in association with ESBL-E acquisition remained.

However, the microbial community structure is less stable among travelers who developed travelers’ diarrhea as the within-subject Bray–Curtis distance was significantly larger among travelers that experienced diarrhea compared to those that did not (coefficient estimate: 0.074, 95% CI[0.03, 0.12]; *P* = .002). There was also a significantly larger within-subject Bray–Curtis distance between travelers who ate at local street food stalls daily (Coefficient estimate: 0.176, 95% CI[0.07, 0.28]; *P* = .001). Upon stratification according to travel destination, this signal remained for travelers eating daily from street food stalls in Southeast Asia (Supplementary Table 2).

### Changes in the taxonomic and functional composition of the microbiome

**Figure 5.**
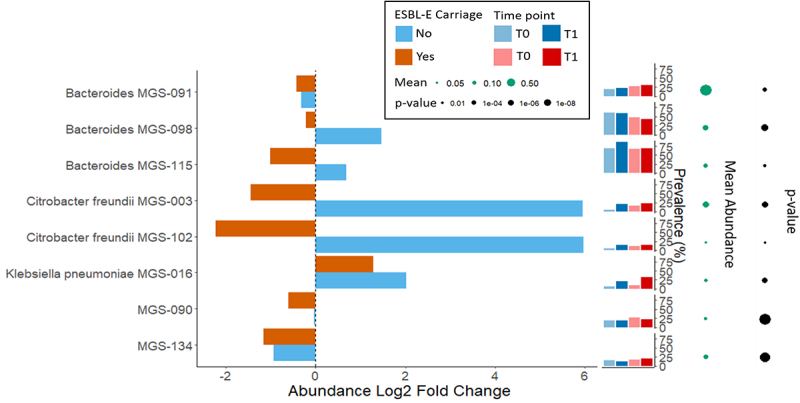
Taxonomic composition shift in association with ESBL-E acquisition. Plot depicting the log2-fold change of abundance, total presence count (prevalence), median relative abundance in total population and p-value of each taxa that significantly changes during travel. Only taxa significantly associated with ESBL-E acquisition were selected for visualization. Differences in pretravel to post-travel composition were tested using zero inflated beta regression with a random effects (ZIBR) statistical model. Analysis was performed with the post-travel abundances as dependent variables and pretravel abundances, ESBL-E acquisition, sex, age, BMI and travelers’ diarrhea as the covariates for both the logistic and beta components.

The shifts in prevalence and relative abundance of individual microbial taxa in association with ESBL-E acquisition were next examined with ZIBR.^23^ Eight MGSs were significantly associated with ESBL-E acquisition during travel (figure 5). Three belonged to the family Enterobacteriaceae (MGS-003 and MGS-102 were classified as *Citrobacter freundii* and MGS-016 as *Klebsiella pneumoniae*), and three belonged to the genus *Bacteroides* (these MGSs did not meet the threshold for species level classification, i.e., <70% of hits to the same species, see Supplementary Juliap). The genes within the remaining two MGSs hits various phyla, and interpretation for theslatter two MGSs should therefore be done with caution as these might be potential artifacts.

MGS-091, the most abundant *Bacteroides* MGS (see Supplementary Figure 1 for baseline relative abundances), had both a 0.42-fold decrease and a 0.33-fold decrease in travelers who did and did not acquire ESBL-E, respectively. *Bacteroides* MGS-098 had a 1.47-fold increase in abundance in travelers who did not acquire ESBL-E, but a 0.21-fold decrease in travelers who acquired ESBL-E. Additionally, MGS-115, the most prevalent *Bacteroides*, had a 0.69-fold increase in travelers remaining ESBL-E-negative and a 1.01-fold decrease in travelers who acquire ESBL-E.

The most pronounced shifts were, however, observed for two MGSs identified as *Citrobacter freundii*. Both *C. freundii* MGS showed similar longitudinal patterns; increasing 5.96 and 5.95 fold, respectively, in travelers who did not acquire ESBL-E while decreasing 2.23 and 1.45 fold in travelers who did. The abundance of MGS-016, identified as *Klebsiella pneumoniae*, has both a 1.28- and 2.02-fold increase in those subjects who did and did not acquire ESBL-E, respectively. However, the increase in its prevalence from 8.9% to 31.7% of subjects is larger in travelers who acquire ESBL-E during travel, compared to 7% to 20.8% of subjects in those who remain negative for ESBL-E.

MGS-090 had a 0.04-fold decrease in travelers who remained negative for ESBL-E, but a 0.6-fold decrease in those who acquired ESBL-E. MGS-134 had a 0.94- and 1.16-fold decrease in travelers who remained negative for ESBL-E and acquired ESBL-E, respectively.

The metabolic profile of the samples was determined by functionally annotating each gene of each MGS and clustering by the KEGG module. Samples were heirarchically clustered by distance to each other, based on the fold changes of each module, but did not cluster by ESBL-E acquisition (Supplementary Figure 2). To examine if short-chain fatty acid production might be protective for ESBL-E acquisition, we next examined the abundances of KEGG Orthology genes involved in butyrate, succinate and propionate production. Neither the baseline abundances of these genes nor the abundances in post-travel samples or shifts in abundance between pre- and post-travel were associated with ESBL-E acquisition (Supplementary Tables 4–6).

## Discussion

The gut microbiome’s role in providing colonization resistance against invading bacteria is of particular relevance in people traveling internationally to countries in which MDR bacteria are endemic. This study aimed to discover which aspects of the microbiome structure – or consortia of bacteria – contribute to colonization resistance against MDR bacteria. Neither the microbial diversity and community structure in pretravel samples nor the longitudinal change of these metrics during travel were predictive of the susceptibility to acquiring ESBL-E. Alternatively, prevalence and abundance changes in individual microbial taxa belonging to the family *Enterobacteriaceae* and genus *Bacteroides* were significantly associated with ESBL-E colonization risk.

Studies focusing on the association between colonization with MDR bacteria and the gut microbiota primarily utilize 16S rRNA gene amplicon methods, which have limitations in their lack of resolution to a species level analysis, and/or cross-sectional studies, which do not capture the longitudinal microbial changes. ESBL-E acquisition in travelers has been researched with meta-transcriptomics,^21^ where reads were mapped to 16S and 23S rRNA databases for identification, and small-scale WMGS on a data set of up to 10 travelers.^22^ There is therefore a limited scope and depth of current research into the role of the microbiome in this field, so in this study, the WMGS of 179 travelers provides a significant contribution to the closure of a major gap in the literature.

In our study, the diversity and community composition did not differ between travelers before travel, or within individuals during travel, in relation to ESBL-E acquisition. Instead, after the development of travelers’ diarrhea, the diversity is significantly reduced and community composition is drastically altered, consistent with the literature.^6,21,22^ Travelers’ diarrhea is known to be associated with an increase in ESBL-E acquisition,^7^ but ESBL-E acquisition without travelers’ diarrhea seemingly has little effect on the microbiome richness and diversity. This is unlike previous studies that associated increased pathogenic bacteria with a reduced microbial richness and altered microbiome in hospital patients.^24^ However, samples collected from hospitalized patients are often confounded by antibiotic use, which reduces the diversity and richness measurements, as well as significantly perturbing even stable microbiomes. The results here are therefore suggesting that ESBL-E can colonize a traveler’s microbiome without perturbation and without causing symptoms, but in those who develop travelers’ diarrhea, it is difficult to determine if ESBL-E colonization precedes the onset of diarrhea.

In this study, MGS both identified as *Citrobacter freundii* had a statistically significant increase in abundance in travelers who did not acquire ESBL-E. Commensal *Enterobacteriaceae*, of which *Citrobacter* is a member, are continuously competing with other commensal or invading members of the family. This is achieved through various mechanisms, such as the secretion of antimicrobial proteins,^25^ the metabolism of nutrients^26^ or competition for oxygen.^27^ These mechanisms may change the host’s susceptibility to colonizing species,^28^ as it must have the necessary pathways to outcompete. Further studies on the interactions between *C. freundii* and ESBL-E in the human guts are required to uncover the mechanisms by which ESBL-E colonization might be inhibited by *C. freundii.*

A statistically significant increase in abundance of two MGS of *Bacteroides* was also associated with travelers who remained negative for ESBL-E, whereas a decrease of these MGS as well as an additional *Bacteroides* MGS was observed in travelers who acquired ESBL-E. The healthy gut microbiome is usually dominated by the strict anaerobe phyla of *Bacteroidetes* (of which *Bacteroides* is a member) and *Firmicutes*, whereas the facultative anaerobes of *Enterobacteriaceae* populate at a much lower proportion.^29^ Interestingly, in a recent cross-sectional study on the association between the fecal microbiota and ESBL-E colonization among 200 healthy volunteers living in rural Thailand, the most profound difference was detected in the phylum Bacteroidetes. In particular, the abundance of *B. uniformis* was significantly lower in ESBL-E carriers as compared to noncarriers.^30^

Various *Bacteroides* species exhibit immunomodulatory effects that are beneficial to the host and can increase the colonization resistance to invading bacteria. A major mechanism of this is the inhibition of host inflammation via the secretion of sphingolipids,^31^ a signaling molecule or outer-member vesicles containing polysaccharide A,^32^ a molecule that induces the production of T cells to suppress inflammation.^33^ Low-grade intestinal inflammation perturbs the gut microbiome and can enhance the colonization of *E. coli*,^34^ so reducing this is vital to maintain colonization resistance to invading bacteria. *Bacteroides* species may have further immunomodulatory effects via the improvement of host macrophage’s phagocytic function^35^ or via an increased xylan degradation,^36^ which has links to an enhanced immune system and improved health.^37,38^ Moreover, *Bacteroides* species can produce a variety of short-chain fatty acids and organic acids, including acetic acid, propionic acid, isovaleric acid and succinic acid. Short-chain fatty acids have been shown to provide colonization resistance against antibiotic-resistant *Enterobacteriaceae*, among others, by triggering intracellular acidification.^39^ To reveal a potential protective role of metabolites, including sphingolipids, xylan breakdown products and short-chain fatty acids, produced by *Bacteroides* species or other members of the microbiome, future studies on the fecal metabolome are warranted.

A major strength of this study is the use of whole metagenome shotgun sequencing as a representation of the total microbiome. The longitudinal nature of the data set additionally strengthens the study as it allows for temporal associations on the effects of travel on the microbiome, with a large sample size to correct for confounding factors. However, metagenomics limits the resolution at which to confidently identify individual strains. Instead, the taxa-specific analysis should be interpreted as where to focus potential future research. This study has a limitation with the sampling method employed, as the transport at room temperature and use of Cary–Blair medium on fecal swabs can increase the microbial richness and diversity^40^ and can lead to increase in abundance of taxa, notable *Enterobacteriaceae* and *Ruminococcaceae*, that can grow at room temperature in the presence of oxygen.^41^ However, as the samples all experienced the same conditions, the effects are not likely to differ between samples; instead, the quantities should not be extrapolated to other sampling methods.

The observations of this study highlight the likely importance of discrete species in the dynamics of ESBL-E acquisition, especially within the family *Enterobacteriaeceae*. The identification of species associated with ESBL-E acquisition necessitates for higher resolution research. The current analyses cannot easily detect how the commensal *Enterobacteriaceae* population is interacting with the invading ESBL-producing *E. coli*, so it would be of significant interest to perform experimental studies to explore the competition between various species and strains within this genus and family. The extent at which each member alters the microbiome’s colonization resistance could be discovered. Alternatively, the knowledge on factors facilitating the elimination of previously acquired ESBL-E is still limited, so research on samples taken on additional time points after travel is worthwhile being examined.

This study emphasizes how the overall microbiota diversity, community structure and functional metabolism play little role in the acquisition of ESBL-producing *Enterobacteriaceae*, instead of highlighting the importance of *Bacteroides* and other members of *Enterobacteriaceae*. The discovery of taxa that may contribute to colonization resistance will aid in the development of intervention strategies to minimize the acquisition and spread of MDR bacteria.

## Methods

### Study population and design

The present study was embedded within the COMBAT study (ClinicalTrials.gov identifier: NCT01676974), a longitudinal multicenter study among 2,001 Dutch international travelers for which the design has been described previously.^42^ Briefly, all travelers provided questionnaire data and fecal swabs before travel, immediately upon return and one month after return. Fecal swabs (Fecal Swab®; Copan, Brescia, Italy) were incubated in tryptic soy broth supplemented with vancomycin (50 mg/L) and after overnight culture, subcultured onto chromID ESBL agar plates (bioMerieux, Marcy l’Etoile, France). All morphologically distinct colonies were identified to the species level using a matrix-assisted laser desorption/ionization time-of-flight mass spectrometry (Bruker Microflex LT, Bruker, London, UK). For all Enterobacteriaceae, antibiotic minimum inhibitory concentrations were measured with the automated susceptibility testing system Vitek 2 (bioMerieux). Phenotypical confirmation of ESBL production was performed by combination disc diffusion tests, according to current national Dutch guidelines.^43^ Remaining feces was biobanked at −80°C for future analyses.

For the present study, we randomly selected paired pre- and post-travel biobanked fecal samples from 190 travelers who visited South Asia, Southeast Asia, North Africa or East Africa and who had not used antibiotics in the three months prior to travel departure. Pretravel samples of 11 travelers were positive for ESBL-E, and both pre- and post-travel samples of these travelers were removed from future analyses in order to study shifts in the microbiome in subjects “at risk” for ESBL-E acquisition. The metadata for the resulting 179 subjects who were negative for ESBL-E and had not taken antibiotics within three months prior to travel are displayed in Table 1.

**Table 1.** Description of travelers included in this study (N = 179) and their travel-associated factors.

	Median (IQR)
**Age (years)**	51.6 (19.65–81.68)
**BMI**	23.74 (17.18–35.67)
	**n/N (%)**
**Sex** Male Female	75 (41.3%) 104 (58.1%)
Antibiotic Use During Travel* No Yes	163 (91.1%) 10 (5.59%)
**Region visited during travel** Northern Africa Eastern Africa Southern Asia Southeast Asia	42 (23.5%) 42 (23.5%) 46 (25.7%) 49 (27.4%)
**Travelers’ diarrhea during travel** No Yes	95 (53.1%) 84 (46.9%)
**Acquired ESBL-E during travel** No Yes	76 (42.5%) 103 (57.5%)
*Numbers do not add up to 179 due to missing values.	

Fecal metagenomic DNA was extracted using protocol Q of the International Microbiome Standards Consortium,^44^ which includes bead-beating (on a FastPrep™ Instrument (MP Biomedicals, Santa Ana (CA), USA) with 0.1 mm zirconium-silica beads (BioSpec Products, Bartlesville (OK), USA)) followed by purification using QIAamp DNA Stool Mini kit columns (Qiagen, Hilden, Germany). A Qubit® fluorometer dsDNA HS Assay (Invitrogen) was used to quantify extracted DNA, and this DNA was stored at −20°C.

Diluted DNA (0.5 ng/uL) was prepared for sequencing with a Nextera Library Prep kit as described previously,^45^ purified using the Agencourt AMPure XP system (Beckman Coulter) and quantified using the Quant-iT PicoGreen dsDNA assay (Invitrogen). 10 nM of DNA from approximately 96 samples was equimolarly pooled (three independent times), for each sequencing lane. Pools were submitted for paired-end 2 × 150 base pair sequencing (llumina NextSeq High-Output platform) with a targeted sequencing depth of 5 million reads per sample. Raw shotgun metagenomic reads have been deposited and released to NCBI SRA under BioProject ID PRJNA688274

### Construction of the nonredundant gene catalog

Samples were processed using the MOCAT software.^46^ Reads were trimmed and filtered using fastx,^47^ with a minimum quality cutoff score of 15 and a minimum length cutoff of 60 bp. High-quality reads were assembled using Soap 2.04^48^ into scaftigs of a minimum length of 500 bp.

Scaftigs underwent *de novo* gene prediction via MetaGeneMark.^49^ Predicted genes were clustered using BLAT by single linkage.^50^ Genes were clustered if there was at least a 95% sequence similarity and at least 90% of the shorter gene was covered. This created a nonredundant gene catalog.

High-quality reads were screened against the nonredundant gene catalog with SOAPaligner from Soap 2.04 and the parameters of a length cutoff of 30 bp, a percent cutoff of 95% identity, a seed length of 30, a maximum number of mismatches of 5, a random assignment of multiple matches and the – M 4 flag indicating for it to find the best hits. Coverage of each gene per sample was calculated using the soap.coverage script (source code available at Ref. ^50^), and a gene abundance table was produced.

### Generation of coabundance gene groups and metagenomic species

The abundance table was analyzed with the canopy clustering algorithm (source code available at^50^) that groups genes by similar abundance patterns across the samples. A canopy was created by grouping genes to a randomly selected gene within a distance of > 0.9 Pearson correlation coefficient and > 0.6 Spearman’s rank correlation coefficient. Multiple canopies were clustered together if their median abundances had a distance of > 0.97 Pearson correlation coefficient. Canopies were considered to be of insufficient quality when containing only 2 genes; at least 90% of their abundance was made up from only 3 samples or were present in less than 4 samples. Canopies of a high quality were identified as coabundance gene groups (CAGs) and CAGs with at least 700 genes contained enough information to potentially be classified as metagenomic species (MGS).

### Taxonomic classification and abundance calculation of MGS

The genes of each MGS were aligned to the blast nucleotide database (blastdb_nt_v5, accessed: January 2020) using BLASTN (v2.6.0+^51^) at a percentage identity of >45% on at least 100 bp and a maximum of 50 hits for each gene were kept. Bacterial taxa that received fewer than 25 hits were filtered out as noise. An MGS was assigned to a species if at least 70% of the total hits matched at a sequence similarity of >95% on at least 100 bp. Parameters were reduced to 60% of hits at >85% similarity to be assigned to a genus.

An MGS was quantified by mapping reads at >95% on at least 100 bp to the top 100 most correlated genes; counts were normalized by the total nucleotide length of these 100 genes and by sequencing depth of the samples. Individual CAG counts were only calculated for samples where at least three genes of that CAG were present.

### Functional profiling

All genes in each MGS were converted into amino acid sequences with Transeq.^52^ Amino acid sequences were orthology assigned to the eggNOG v5.0 database^53^ (accessed: January 2020) by eggNOG-mapper.^54^ KEGG annotations were selected from the results, and pathways were grouped into KEGG modules for downstream analysis. KEGG abundance per sample was calculated as the sum of the MGS abundances in which the KEGG module was detected.

Additionally, the initial nonredundant gene catalog was annotated to KEGG Orthology (KO) and KOs belonging to butyrate, succinate and proprionate production were selected. Total microbiome abundances were calculated from the gene catalogs’ equalized gene abundance table, and by summing every instance, a KO was annotated to a gene in each traveler.

### Microbiome richness, diversity and community structure measurements

Statistical analyses were carried out in R on the entire study population as well as stratified according to travel destination. Alpha diversity measurements of species richness, Chao1 index and effective Shannon index were calculated using Vegan.^55^ The dissimilarity in the microbial community structure (beta diversity) between samples was calculated by means of the Bray–Curtis dissimilarity index. Associations between the microbial community structure and metadata were determined using Envfit from the package Vegan.

To examine changes in microbial richness and diversity over time, the delta in alpha-diversity (intra-individual change in microbial richness/diversity between pre- and post-travel samples) was calculated. To examine the (in)stability of the microbial community structure, the within-subject Bray–Curtis distance between pre- and post-travel samples was calculated.

Univariate analysis consisted of the t-test or Mann Whitney U-test, depending on normality as determined by the Shapiro-Wilk normality test, which was applied to examine associations between ESBL-E acquisition and microbial richness, diversity or SCFA abundance at baseline, as well as longitudinal changes in richness (observed species, Chao1), diversity (effective Shannon), SCFA abundance and community structure (Bray Curtis dissimilarity) measurements. Multivariate analysis consisted of fitting the data to a linear regression model, with the diversity measurement as the outcome and ESBL-E acquisition as the main covariate of interest, while adjusting for the following potential confounding factors: age (years), sex (male/female), BMI, travelers’ diarrhea (yes/no) and food consumption at food stalls (never/sometimes/daily).

### Taxonomic and functional statistical analysis

To reduce the number of comparisons, taxa or KEGG modules present in <10% of samples or in which the relative abundance in the top 10% of samples was below 0.1% were removed from further taxonomic and functional analysis.

Zero-inflated two-step beta regression (ZIBR) analysis^23^ was carried out on all remaining taxa; except for taxa absent in less than 10 samples as this is a prerequisite of the zero-inflated aspect of the model. The model was fit using the post-travel CAG as the dependent variable and its pretravel abundance, ESBL-E acquisition, age, sex, BMI and travelers’ diarrhea as covariates for both the logistic and beta components of the model. The time point variable was set to 1 due to the study containing only two time points, and the Gaussian quadrature points was set to 30. Analysis was carried out on all samples collectively and repeated for each travel destination separately.

A negative binomial generalized linear model^56^ was used for the remaining taxa detected in the majority of samples (i.e. absent in less than 10 samples). Post-travel CAG abundance was predicted against ESBL-E acquisition while using pretravel CAG abundance, age, sex, BMI and travelers’ diarrhea as additional covariates. Negative binomial generalized linear models were also used to study changes in KEGG module abundance in association with ESBL-E acquisition.

## Supplementary Material

Supplemental Material

## Data Availability

Raw shotgun metagenomic reads have been deposited to NCBI SRA under BioProject ID PRJNA688274, and each is titled ‘WGS of Homo sapiens: Dutch traveler stool’. The software packages used in this study are free and open source. Analysis scripts are available from MD on reasonable request.
