# Supplementary figures and images for: Enterobacteriaceae and Bacteroidaceae provide resistance to travel-associated intestinal colonization by multi-drug resistant Escherichia coli
Source: Gut Microbes. 2022 Apr 7;14(1):2060676. doi: 10.1080/19490976.2022.2060676 (PMC8993065; doi:10.1080/19490976.2022.2060676)

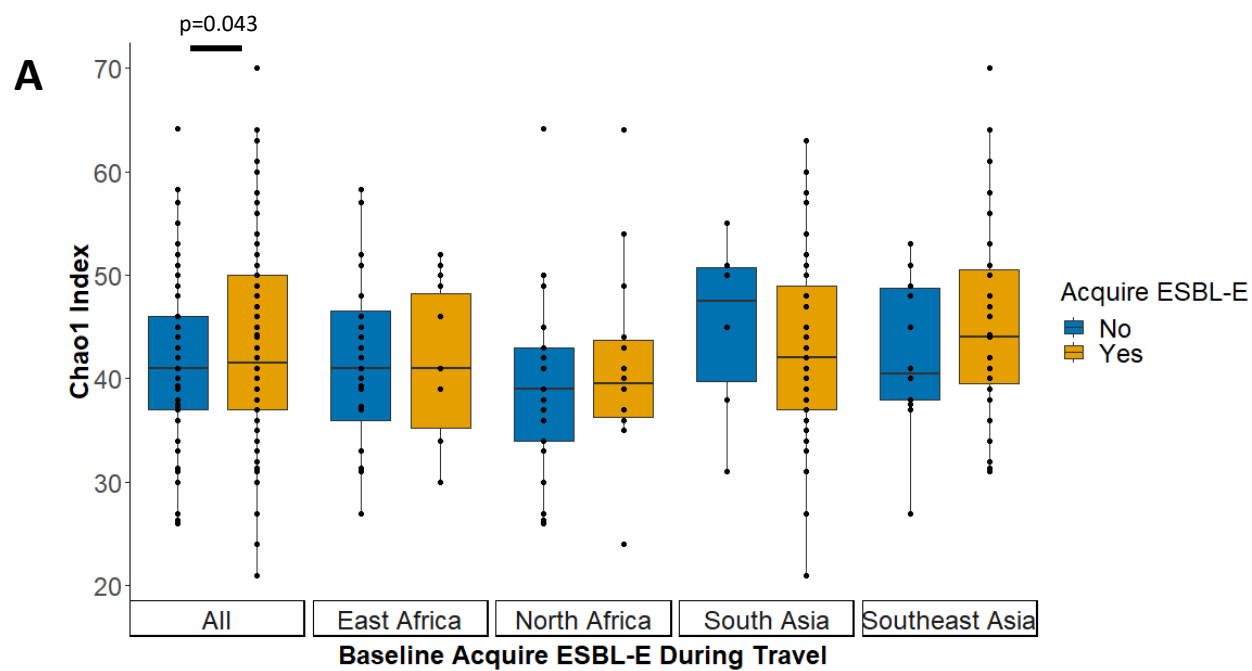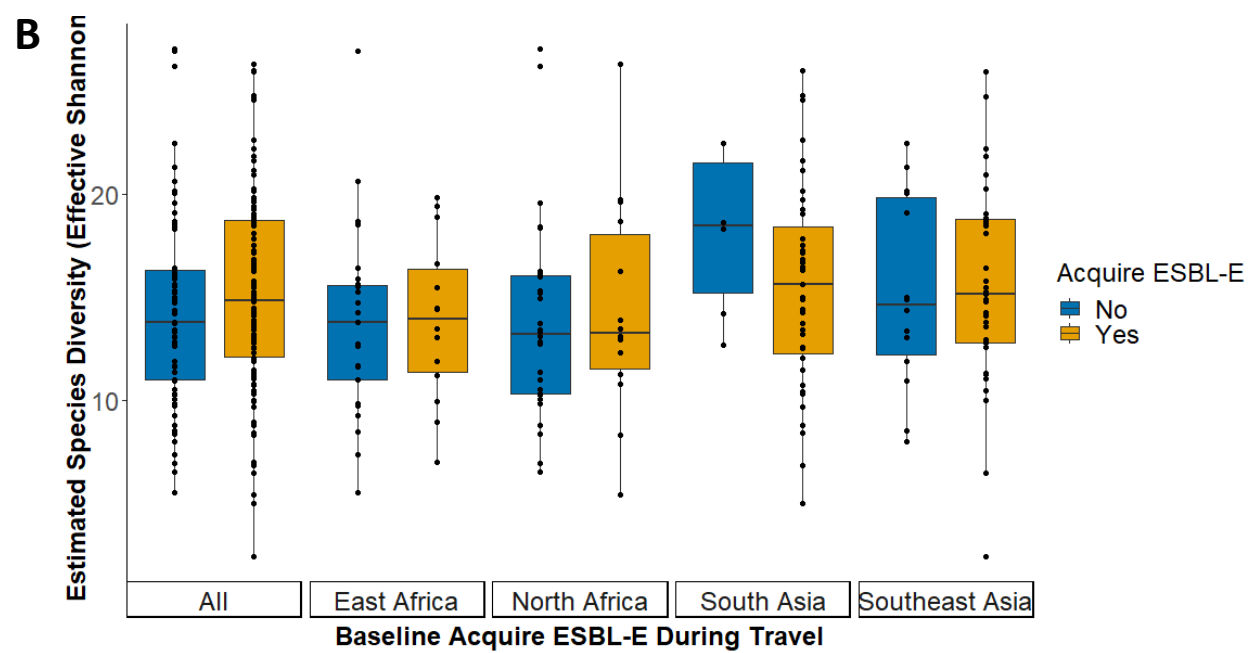

Supplement: Supplemental Material [file KGMI_A_2060676_SM1727.zip › Supplementary Figure 1.pdf]

KEGG Pathway

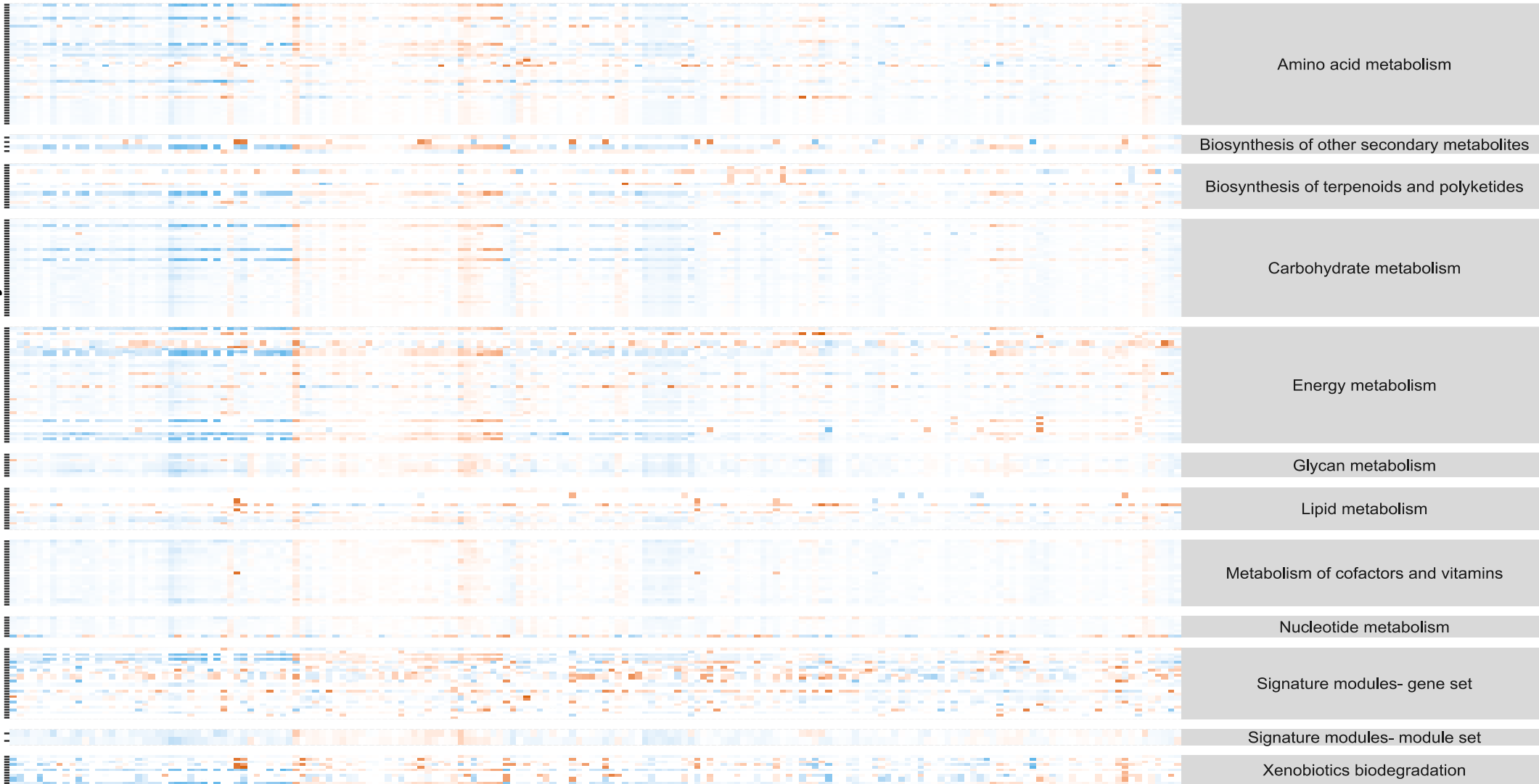

Log2 Fold Change

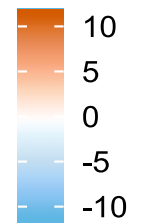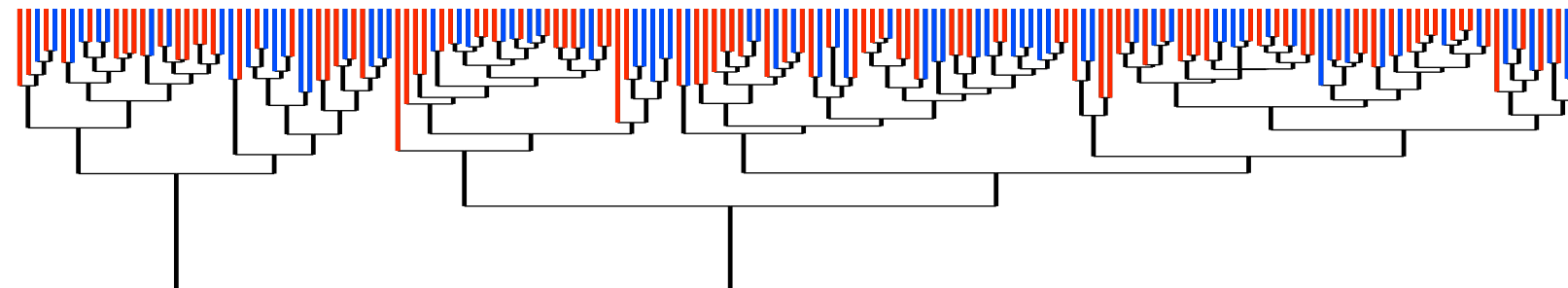

ESBL Carriage

Yes —

No —

Supplement: Supplemental Material [file KGMI_A_2060676_SM1727.zip › Supplementary_Figure_2.pdf]
